# Pathological fractures and the perils of purchasing steroids online

**DOI:** 10.1016/j.radcr.2024.09.025

**Published:** 2024-10-14

**Authors:** Danielle C. Thor, Valerie Rome

**Affiliations:** a Jefferson Health New Jersey, Stratford, NJ 08084, USA; b Rowan-Virtua School of Osteopathic Medicine, Stratford, NJ 08084, USA

**Keywords:** Glucocorticoids, GIOP, Steroids, Pathological fractures, Steroid dependence, PM&R, Clinical imaging

## Abstract

Exogenous steroids are a staple of modern medicine, with utility in a variety of direct and supplemental treatment modalities. However, all pharmaceuticals are undoubtedly coupled with unique side effect profiles, and steroids are no exception. In the case presented, the assessment and management of a 36-year-old male who inappropriately self-administered high-dose steroids for prolonged periods of time is reviewed. In doing so, attention is drawn to both the impact of glucocorticoid-induced osteoporosis and the greater risk of online availability of these powerful medications.

## Introduction

Since their inception in the early 1950s, exogenous glucocorticoids, or steroids, have been utilized throughout medical practice [1]. Examples include obstructive pulmonary diseases, autoimmune or rheumatological disorders, and specific infections with resultant inflammatory disorders [2]. Although they maintain a diverse array of therapeutic indications, glucocorticoids are not benign medications. Adverse effects associated with steroid usage range from significant depression of one's immune system to cardiovascular dysfunction, muscular atrophy, and osteoporosis with potential pathological fractures [2]. Extended steroid usage can also be coupled with steroid dependence syndromes, where patients struggle to taper off glucocorticoids due to recurrence of symptoms or psychological dependence on pain reduction from their anti-inflammatory effects [2]. However, in controlled situations with appropriate clinician supervision and tapering, many of these side effects can be prevented.

Glucocorticoid-induced osteoporosis (GIOP) is recognized as the most common cause of secondary osteoporosis, with or without an iatrogenic origin [3]. In the case presented here, a 36-year-old male unfortunately develops a steroid dependence over several years of chronic back pain management with intermittent steroid prescriptions. He is ultimately able to employ the inconsistencies within current telehealth systems and/or digital pharmacies to obtain and self-administer increasing doses of exogenous steroids. Through this novel drug-seeking channel, he inevitably developed GIOP with associated pathological fractures.

## Case description

A 36-year-old male with no pertinent medical history but prior surgical history of anterior cervical discectomy and fusion to cervical vertebrae 3-5 years prior to arrival presented to the emergency department for evaluation of lower back pain. He stated that he works as a truck driver and that he felt a “pop,” followed by a cracking sensation, while attempting to make a wide turn with his truck. Upon examination, the patient described acute-on-chronic lower back pain worsening over the past week prior to arrival, as well as urinary incontinence, clonus, unsteady gait, and bilateral lower extremity weakness without associated hip or leg pain. At the time of admission, the patient stated that he had been taking high-dose steroids for several days prior to arrival but was unable to identify why he was prescribed them.

**Fig. 1:**
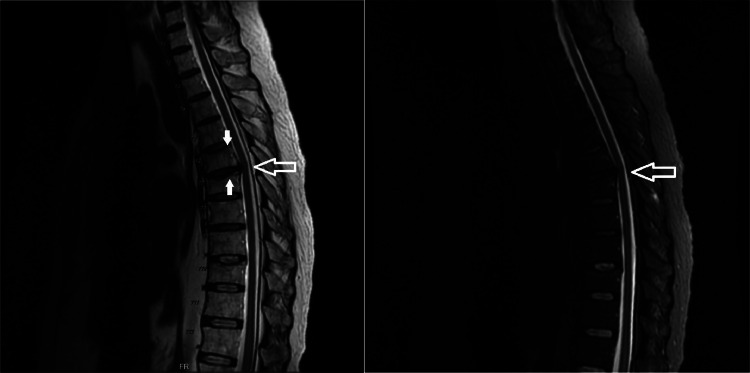
Key images from the MRI of the thoracic spine, with focus placed on the pathological fractures of T6 and T7 (shaded arrows) and degenerative disc disease at the same spinal level causing mild central canal stenosis (nonshaded arrows).

**Fig. 2:**
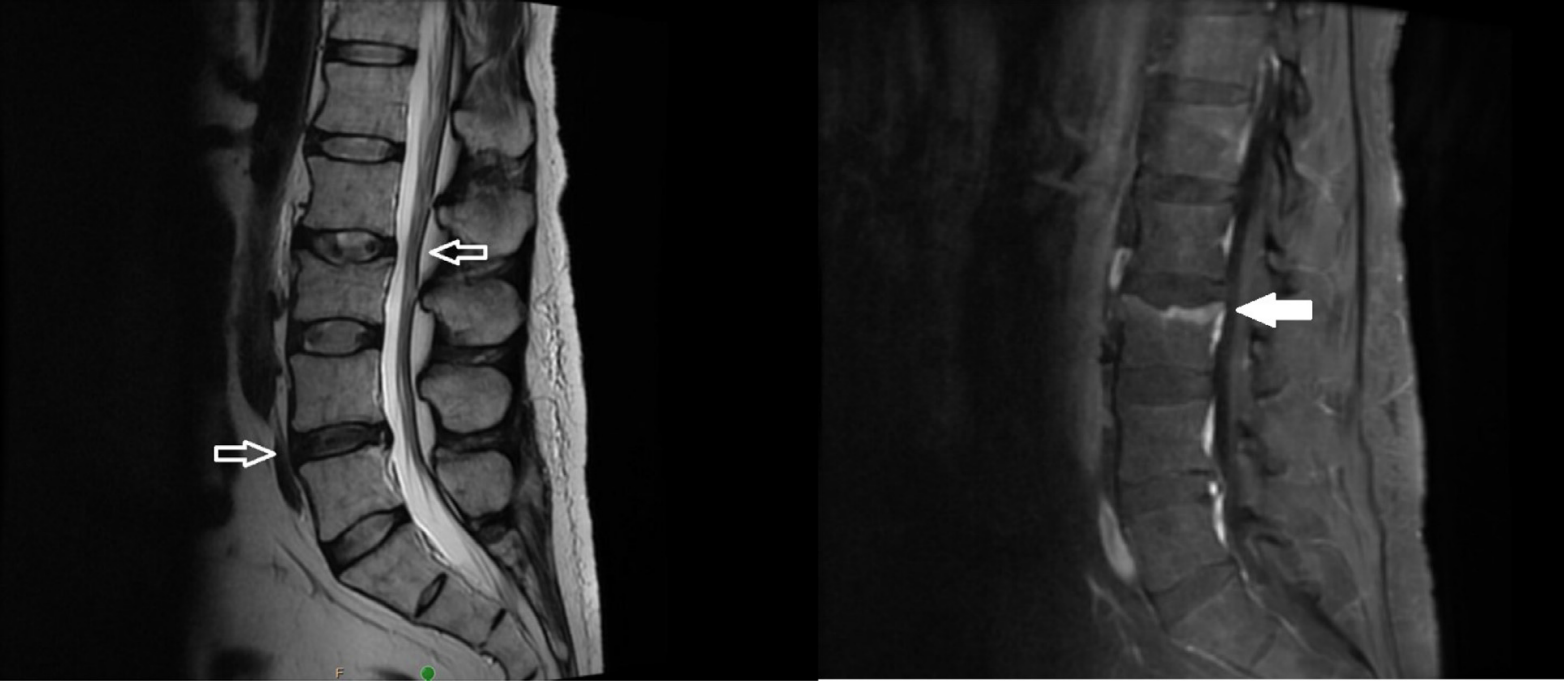
Key images from the MRI of the lumbar spine, with focus placed on the anterior disc protrusion at L4-L5 and the posterior disc protrusion at L2-L3 (nonshaded arrows), as well as the chronic end-plate fracture at L3 (shaded arrow).

The patient was found to be hemodynamically stable throughout his admission. His physical exam was notable for a classic moon facies presentation with truncal obesity and striae. His lab work on admission was notable for an elevated lactate, hyperglycemia with glucosuria, and mild leukocytosis. Magnetic resonance imaging (MRI) without contrast of the length of the spine was obtained and the cervical portion was otherwise unremarkable. However, MRI of the thoracic spine revealed superior endplate compression fractures involving T6, T7, and T10 vertebrae with edema visualized at all 3 levels concerning for subacute fractures, as well as degenerative disc with mild spinal canal stenosis at T6-T7 (Fig. 1). Additionally, MRI of the lumbar spine revealed a chronic-appearing superior endplate fracture of L3 with mild vertebral body height loss, mild spinal canal stenosis at L2-L3, and small-to-moderate broad-based disc protrusion at L4-L5 causing mild mass effect on the anterior thecal sac (Fig. 2).

**Fig. 3:**
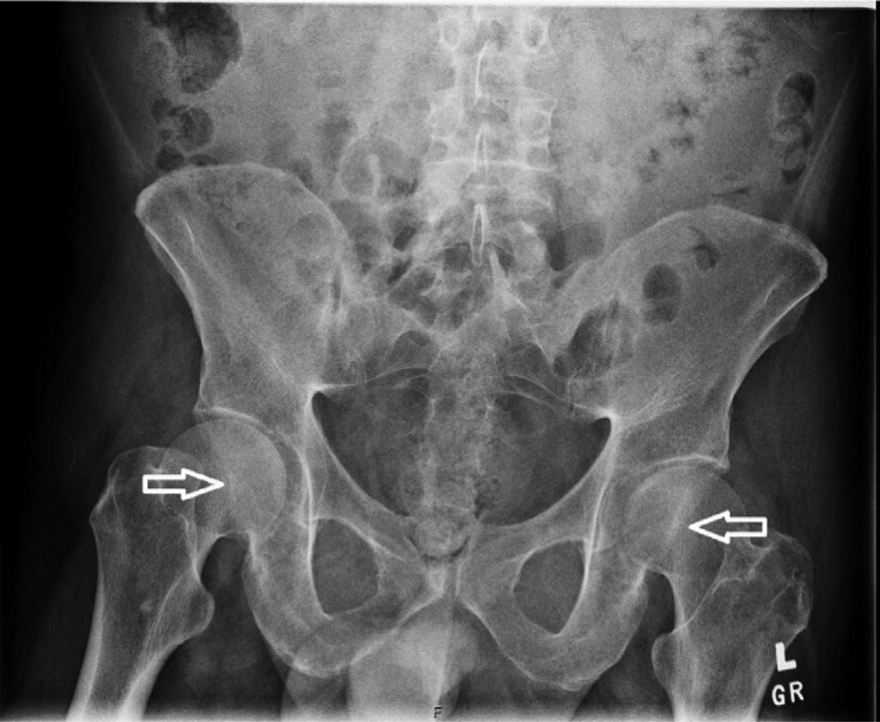
Key image from bilateral hip and pelvis X-rays with mild sclerosis of the femoral heads originally concerning for early-stage avascular necrosis (nonshaded arrows).

**Fig. 4:**
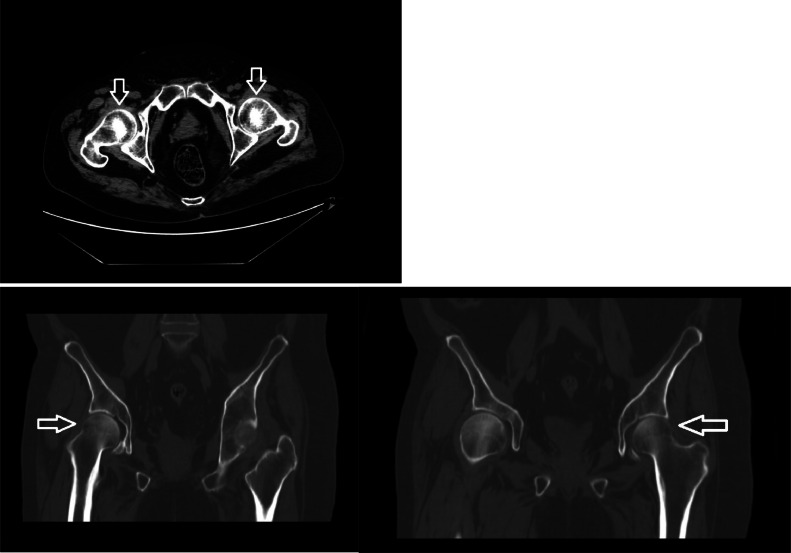
CT of the bony pelvis without contrast notable for mild, increased sclerosis concerning for bilateral, early-stage avascular necrosis of the femoral heads (nonshaded arrows).

Furthermore, bilateral hip and pelvis X-rays were obtained, which were concerning for concurrent early-stage avascular necrosis of the bilateral femoral heads (Fig. 3). These findings were later confirmed with dedicated computed tomography (CT) imaging of the bony pelvis (Fig. 4). In-house neurosurgery and orthopedic surgery were consulted in response to these findings, however, neither practice recommended an acute surgical intervention on this admission.

Further discussion with the patient revealed a workplace accident approximately 3 years prior to admission, which resulted in longstanding lower back pain. The patient believed he derived the most symptomatic benefit from his original steroid prescription versus prior encounters for physical therapy or other pain management modalities. He began to utilize various telemedicine clinicians and a series of online pharmacies to obtain multiple prescriptions of prednisone and supplemental methylprednisolone dose packs, which he would self-administer with intermittent increases in dosing over time. In addition, the patient noted periods of withdrawal between prescriptions, including intolerable anxiety, palpitations, and diaphoresis, thereby encouraging his prescription-seeking behavior. At the time of admission, the patient was maintaining his steroid dependence with a minimum of 50 milligrams of prednisone daily.

Extensive education was provided to the patient about the extent of his pathological fractures, with his steroid dependence serving as a likely source. The patient was ultimately agreeable to an extended steroid taper and subsequent rehabilitation. He received a fitted thoracolumbar sacral orthosis (TLSO) and was eventually discharged to an acute rehabilitation facility.

## Discussion

In individuals under 50 years old, GIOP is the most common cause of secondary osteoporosis [3]. This adverse effect arises from the innate nature of glucocorticoids as combination suppressors of osteoblast function and promoters of osteoblast activity. This synergistic mechanism results in an increase in bone resorption and an overall reduction of bone mineral density, thereby generating the opportunity for secondary osteoporosis and subsequent pathological fractures [4]. In the case of this 36-year-old male, his continued, unauthorized use of increasing doses of glucocorticoids resulted in a textbook presentation of GIOP with subsequent pathological fractures of the thoracic vertebrae as well as avascular necrosis of the bilateral hips.

Pathological fractures in GIOP typically occur in the vertebral column, hips, or wrists, but are not limited to these presentations [5]. Appropriate imaging typically begins with plain radiographs of the affected area to determine the extent of the fracture(s) with minimal cost or radiation exposure [6]. However, given the presence of “alarm symptoms” in this case, or urinary incontinence, clonus, and bilateral low extremity weakness, a clinical concern for cauda equina syndrome was present and more detailed upfront imaging was thereby warranted. The best method for first-time radiological evaluation of cauda equina patients remains urgent MRI imaging, followed by rapid neurosurgical or orthopedic surgery intervention as indicated [7].

Due to the potentially diverse nature of pathological fracture locations and subsequent pain profiles, the examination of any patient with such fractures requires additional evaluation for potential distracting injuries. The case presented is notable for the additional finding of incidental bilateral avascular necrosis of the femoral heads. This finding presented otherwise asymptomatically in this patient, as he was distracted by his original back pain and other “alarm symptoms,” as noted above. Additionally, although plain X-rays are indicated in the initial evaluation of avascular necrosis, early stages of the disease may not yet present with this modality [8]. Dedicated MRI imaging is preferred in earlier stages for its high sensitivity in predicting bone edema [9]. However, given the extent of this patient's musculoskeletal disease and radiological exposure on this admission, his CT of the bony pelvis and clinical picture sufficed for diagnosis of his concurrent avascular necrosis. Thorough evaluation of all patients suspected to have GIOP through modalities such as dual-energy X-ray absorptiometry (DEXA) scanning, targeted x-ray imaging, or more advanced imaging, such as CT or MRI, ultimately remains necessary for improved detection of distracting injuries [5,10].

As the prevalence of prescription steroids expands over time, the prevention and management of any resulting GIOP is crucial for all practicing clinicians. Continual assessments of patient's health literacy and the formation of strong patient-provider relationships when possible are necessary to avoid patient confusion and provide reasonably informed consent about the benefits and risks of steroid exposure [[11], [12]]. Diligent risk assessments and monitoring of all patients prescribed extended steroid courses should be reiterated and include discussions of why to avoid “online shopping” and/or multiple telehealth for excessive prescriptions. Bisphosphonates, such as alendronate and zoledronic acid, may also be considered to mitigate bone loss in situations where extended steroid usage is unavoidable [3]. Lastly, an understanding of the potential for distracting injuries is necessary in the setting of newly-recognized steroid abuse [5,10]. Although this case is limited to a single-patient report, it is strengthened by its combined ability to reiterate the known threats of excessive steroid use and recognize a potentially novel channel for prescription-seeking behavior through the inconsistencies of under-regulated online pharmacies and the rising numbers of unconnected telehealth providers.

## Conclusions

Glucocorticoid-induced-osteoporosis (GOIP) with or without pathological fractures is a critical component in the formation of a differential diagnosis for any individual who may be exposed to excessive exogenous steroids. Through the case presented, attention is drawn to both the possibility of steroid abuse through online pharmacies and unregulated telemedicine, as well as the need for a thorough evaluation and strategic treatment design in the setting of GIOP.

## Patient consent

As the Corresponding Author of Eon Publishing, I confirm that written, informed consent was obtained from the patient(s) mentioned in this paper/case report.

The patient was provided with a thorough explanation of the purpose and nature of the publication, and they voluntarily agreed to participate in the publication of their case details, including any personal information and images.

It was clearly communicated to the patient that their identity would be protected, and all identifying information would be removed or anonymized in the published article. The patient was allowed to ask questions and express any concerns before providing their consent.

The patient was assured that their decision to participate or decline participation would not impact their current or future medical care. They were informed that they have the right to withdraw their consent at any time before publication and that their confidentiality would be maintained regardless of their decision. The patient was also informed that their case may be used for educational or research purposes, and they expressed their understanding and acceptance of this.

A copy of the written consent form has been retained in my records, as per the ethical guidelines and policies outlined by your publisher. However, copies of the consent form have not been provided to the journal unless specifically requested under exceptional circumstances.

I hereby confirm that the patient's consent was obtained in accordance with the ethical standards and regulations governing the publication of case reports and patient information.
